# Metabolite Analysis of *Camellia oleifera* Fruit Pericarp Using UPLC-MS/MS: A Comparative Study of Three Oil Tea Varieties

**DOI:** 10.3390/ijms252211973

**Published:** 2024-11-07

**Authors:** Shengqun Chen, Jiajuan Xu, Shuang Qu, Xia Jiang, Gang Wang

**Affiliations:** 1 Key Laboratory of National Forestry and Grassland Administration on Biodiversity Conservation in Karst Mountainous Areas of Southwestern China, Guizhou Academy of Forestry, Guiyang 550005, China; squnchen@163.com (S.C.); rrylxjj@163.com (J.X.); sjfeng@gzu.edu.cn (X.J.); 2 Guizhou Liping Rocky Desertification Ecosystem Observation and Research Station, Liping 556200, China

**Keywords:** *Camellia oleifera*, flavonoid, UPLC-ESI-MS/MS, metabolites

## Abstract

*Camellia oleifera*, a widely cultivated woody oil crop, holds economic significance because of its ability to grow without encroaching on cultivated land. The pericarp of *C. oleifera* is abundant in flavonoids and phenolic acids, which offer significant nutritional benefits. This study used metabolomic technology (UPLC-ESI-MS/MS) to discern metabolite variances in the pericarp of three *C. oleifera* types (COT, BFOT, and SFOT) during the maturity stage and subsequently analyzed and compared them. A total of 1117 metabolites were detected in the study, including 277 flavonoids, 221 phenolic acids, 108 lipids, 93 amino acids and their derivatives, 83 organic acids, 59 nucleotides and their derivatives, 57 alkaloids, 52 lignans, 44 tannins, 23 terpenoids, and 100 miscellaneous metabolites (such as sugars, alcohols, vitamins, and other unclassified substances). Clustering and PCA analyses revealed distinct separation of COT, BFOT, and SFOT, indicating variances in metabolites within the pericarp peels of these three *C. oleifera* types. KEGG enrichment analysis demonstrated that 143 shared differential metabolites were primarily associated with amino acid biosynthesis. These findings are expected to significantly enhance the current knowledge of the *C. oleifera* pericarp and pave the way for future development and use efforts.

## 1. Introduction

*Camellia oleifera*, a member of the Theaceae family, is a woody tree that has been cultivated in China for over 2300 years. Along with oil palm, olive, and coconut, it is recognized as one of the four major woody edible oil plants globally [1]. The seeds of *C. oleifera* are primarily used for oil extraction, yielding a high-quality edible oil with an unsaturated fatty acid content ranging from 85% to 92%, surpassing that of oleic acid at 80%. In addition, *C. oleifera* oil contains vitamin E, squalene, sterols, polyphenols, carotene, camellia glycosides, saponins, and minerals, which serve as functional nutrients. Renowned for its high levels of unsaturated fatty acids and catechins, *C. oleifera* seed oil boasts notable whitening and antioxidant properties [2]. Tea polyphenols are natural antioxidants renowned for their significant effects in scavenging free radicals and safeguarding cell membrane structure. In addition, Camellia saponins exhibit anti-permeability, anti-inflammatory, analgesic, and anti-cancer properties [3,4].

The mature pericarp of *Camellia* is typically adapted to protect the seed from external hazards, and most previous studies have focused on its thickness and composition [5]. The pericarp of *C. oleifera* was found to consist of cellulose, hemicellulose, lignin, and a small percentage of other components [5,6]. The recalcitrance of the lignified pericarp increases the processing cost of seed accessibility, and the pericarp is considered an economically significant structural feature of Camellia species [5]. The pericarp of *C. oleifera* contains phenols with high antioxidant activity, whereas *C. oleifera* cattail extract demonstrates high safety and efficacy in managing dyslipidemia, with pronounced therapeutic and preventive effects against diet-induced obesity [7]. By-products generated during *C. oleifera* oil processing, such as pericarp, shell, and cake, comprise over 80% of the total oil biomass resources. These by-products, which are abundant in lignin, anthocyanin, theophylline, and pentose, were formerly used as fuel or discarded waste. However, they are now frequently employed in the production of various chemical products, including tannin extract, furfural, xylitol, activated carbon, potassium carbonate, and pyrophosphate [8,9].

Ultra-performance liquid chromatography-tandem mass spectrometry (UPLC-MS/MS) is an advanced analytical technique that combines the high separation efficiency of UPLC with the sensitivity and specificity of mass spectrometry (MS). This technology is becoming increasingly important in fields such as pharmaceutical analysis, biochemistry, food safety, and environmental monitoring [10]. Compared to traditional high-performance liquid chromatography (HPLC), UPLC-MS/MS offers improved sensitivity, greater specificity, higher throughput, and faster analysis, making it a critical tool in pharmaceutical analysis [11]. The comprehensive utilization of the *C. oleifera* industry is pivotal for expanding technology, products, and value chains. While prior studies have predominantly focused on the antioxidant activity within the pericarp of *C. oleifera* [8,9], there has been no prior detection of metabolites in this part of the plant. Investigating valuable compounds pertinent to human health in the pericarp of *C. oleifera* could increase interest in its use. Therefore, the study employed metabonomics to detect metabolites across different varieties of *C. oleifera*. This study offers valuable insights into the comprehensive utilization and development of the *C. oleifera* pericarp.

## 2. Results

### 2.1. Quality Control Analysis

**Figure 1:**
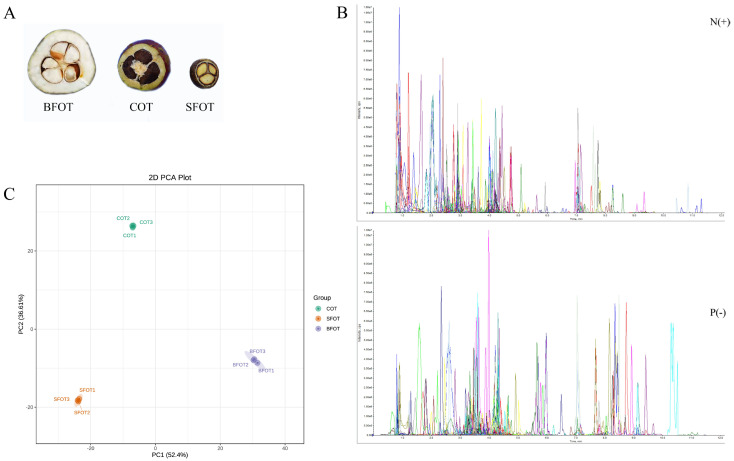
(**A**) Cross-sectional display of three of *C. oleifera* fruits; (**B**) TIC overlap plot for QC sample mass spectrometry detection (N: Negative ion mode, P: Positive ion mode); (**C**) PCA analysis.

Figure 1B illustrates the outcomes of quality control (QC) sample detection in both positive and negative ion modes. The instrument demonstrated remarkable stability in its detection results throughout the mass spectrometry process, reflecting a high degree of consistency across the sample measurements. The peaks generated by various compounds in the two ion modes signify the presence of multiple metabolites within the samples. The extensive range of metabolite coverage is evidenced by the wide distribution of peaks, spanning a detection time range of approximately 0 to 12 min. The presence of multiple distinct, sharp peaks, particularly within the 1 to 10 min interval, indicates high-resolution metabolite detection. The mass spectrometry analysis in both positive and negative ion modes showcases excellent stability and consistency, thereby ensuring reliable sample detection results, comprehensive metabolite coverage, and high resolution. Figure 1C employs PCA to identify the metabolites detected in the pericarps of the three *C. oleifera* varieties. The contribution rates of PC1 and PC2 reached 52.40% and 36.61%, respectively, effectively differentiating the three *C. oleifera* varieties and indicating notable variations in metabolite composition among the pericarps. The biological replicates exhibited a high degree of clustering, demonstrating strong reproducibility within the sample groups. In conclusion, the quality control measures employed in this experiment were effective, ensuring the accuracy and stability of the detection data and providing a robust foundation for further analysis of metabolite differences within the samples.

Figure 1A illustrates the cross-sections of BFOT, COT, and SFOT fruits at the stage of ripening. The BFOT fruits were observed to be the largest in size, with the thickest skin, while the SFOT fruits were the smallest, exhibiting the thinnest skin. We employed non-targeted metabolomics to identify metabolites in three sample groups, resulting in the detection of 11 Class I metabolites, for a total of 1117 metabolites (Class II). These included 277 flavonoids, 221 phenolic acids, 108 lipids, 100 other compounds (such as sugars, alcohols, vitamins, and unclassified substances), 93 amino acids and their derivatives, 83 organic acids, 59 nucleotides and their derivatives, 57 alkaloids, 52 lignans and coumarins, 44 tannins, and 23 terpenes (Table 1 and Table S1). Notably, flavonoids constituted the most abundant group, followed by phenolic acids.

**Figure 2:**
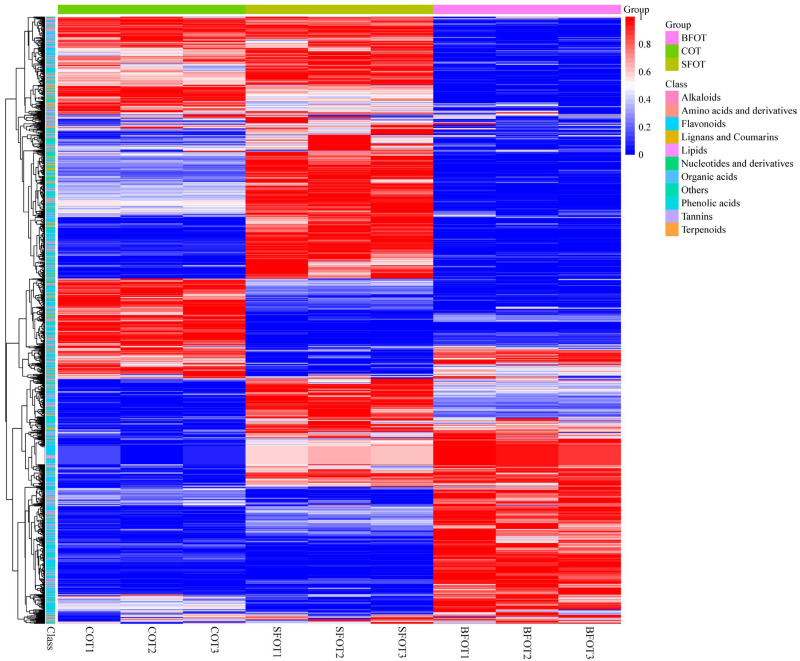
A clustered heatmap showcasing all metabolites, with sample names depicted along the horizontal axis and metabolites along the vertical axis. Red indicates elevated metabolite levels, whereas blue indicates lower levels. The subsequent section details the PLS-DA analysis of three *C. oleifera* fruit pericarps.

The cluster heatmap (Figure 2) illustrates variations in metabolite expression levels across the three types of *C. oleifera* pericarp, indicating significant differences in metabolite expression among them. Compared with the common COT, the concentrations of flavonoids, phenolic acids, and lipids in SFOT and BFOT were lower. Furthermore, BFOT exhibited higher expression levels of most amino acids and their derivatives, lignans and coumarins, alkaloids, and organic acids than COT and SFOT, with SFOT displaying the lowest expression levels. These results underscore the distinct differences observed among the pericarps of the three *C. oleifera* fruit varieties.

### 2.2. Partial Least-Squares Discriminant Analysis (PLS-DA)

**Figure 3:**
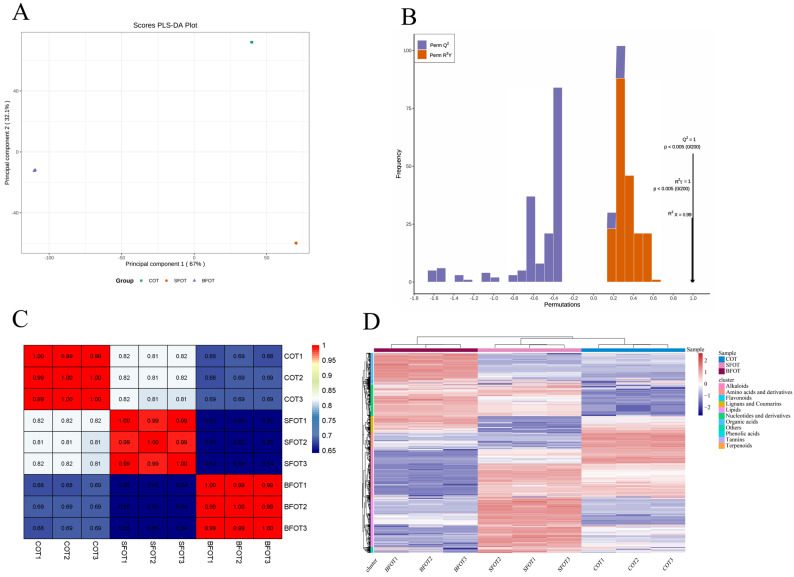
(**A**,**B**) The PLS-DA permutation analysis, where R2X and R2Y represent the model interpretation rate, and Q2 indicates the model’s predictive ability; values closer to 1 indicate greater stability and reliability. (**C**) The heat map of the sample correlation for metabolite profiling. (**D**) Hierarchical clustering heat map of metabolite. The heatmap illustrates the relative abundance of metabolites across different *C. oleifera* varieties (COT, SFOT, BFOT). The color gradient in the heatmap represents the intensity of metabolite abundance, where red indicates higher abundance (positive values) and blue indicates lower abundance (negative values). Sample Grouping (Top Color Legend); Pink: COT (*Camellia oleifera* type). This figure highlights the variation in metabolite profiles across different varieties, emphasizing distinct clusters based on metabolite types and their relative abundance in each sample group.

The results of the PLS-DA analysis revealed distinct metabolic differences among the pericarp samples of the three *C. oleifera* (COT, BFOT, SFOT) (Figure 3A). These differences are characterized by significant variations in the metabolite profiles, particularly those of secondary metabolites such as flavonoids, phenolic acids, and terpenoids. These metabolites are known to play critical roles in plant defense and environmental adaptation, suggesting that metabolite composition may be closely linked to genetic or environmental factors affecting each cultivar. The PLS-DA model (Figure 3B) was statistically significant (*p* < 0.05), with high values for Q^2^ = 1, R^2^Y = 1 and R^2^X = 0.99. These values confirm that the model has excellent predictive ability and explanatory power, effectively capturing the variance in metabolite composition between varieties. The consistency observed within biological replicates (Figure 3C) further supports the reliability of the data. In addition, hierarchical clustering analysis (HCA) based on relative metabolite content (Figure 3D) showed significant differences in most of the metabolites detected, providing further validation of the metabolite profiling and PLS-DA results.

### 2.3. Differential Metabolite Screening

**Figure 4:**
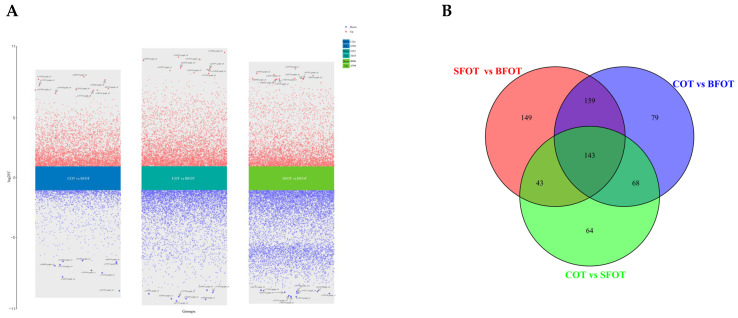
Volcano and Venn maps illustrate differential metabolites, with (**A**) depicting the volcano map and (**B**) displaying the Venn diagram.

We employed variable projection in portance (VIP) ≥ 1 and a difference multiple (FC) ≥ 2 or ≤0.0510 as criteria to discern differential metabolites. The significant differences in metabolites among the three types of *C. oleifera* pericarp are detailed in Supplementary Table S2. The screening outcomes of the differential metabolites (DAMs) in the pericarp of COT, SFOT, and BFOT were visualized using volcano and Wayne diagrams (Figure 4). The volcano plot revealed 318 distinct DAMs in COT vs. SFOT (190 down-regulated, 128 up-regulated), 449 DAMs in COT vs. BFOT (303 down-regulated, 146 up-regulated), and 494 DAMs in SFOT vs. BFOT (383 down-regulated, 111 up-regulated). Flavonoids and phenolic acids predominated among the identified DAMs across the three groups. Through comparative analysis, we identified 143 common DAMs among the diverse fruit peels of *C. oleifera* (Figure 4B).

DAM analysis of COT, SFOT, and BFOT revealed significant differences among certain metabolites (Figure 4A). In the comparison between COT and SFOT, notable differences were observed in metabolites such as epitheaflavic acid-3-O-gallate, 1-O-caffeoyl-4-O-galloyl-ß-D-glucose, quercetin–3–O–(2″–O–arabinose) rutinoside, 6-hydroxykaempferol-3,6-O-diglucoside–7–O-glucuronic acid, granatin A, monogalloyl-glucose, cis-coutaric acid, 2-phenylethanol, procyanidin B3*, and phenylethanolamine. Conversely, in the comparison between COT and BFOT, significant differences were noted in metabolites such as kaempferol−3–O–(6″–rhamnosyl–2″–gucosyl) glucoside (camelliaside A), quercetin–3–O–rutinoside–7–O–rhamnoside, L-ascorbic acid (vitamin C), granatin A, 6-hydroxykaempferol-3,6-O-diglucoside-7-O-glucuronic acid, kaempferol-3-O-glucorhamnoside, 1-O-p-coumaroylquinic acid, coniferyl alcohol–4–O–glucoside (coniferin), phloretin–2″–O–(6″–O–xylosyl)glucoside, and quercetin–3–O–rhamnoside (quercitrin). Moreover, significant differences were observed in SFOT vs. BFOT for L-ascorbic acid (vitamin C), procyanidin B3*, gambiriin A1, 3,4,5-tri-O-galloylshikimic acid, phenylethanolamine, neochlorogenic acid (5-O-caffeoylquinic acid)*, chlorogenic acid (3-O-caffeoylquinic acid)*, 1-O-p-coumaroylquinic acid, ferulic acid-4-O-glucoside, and quercetin-3-O-rhamnoside (quercitrin).

### 2.4. KEGG Annotation and Enrichment Analysis

**Figure 5:**
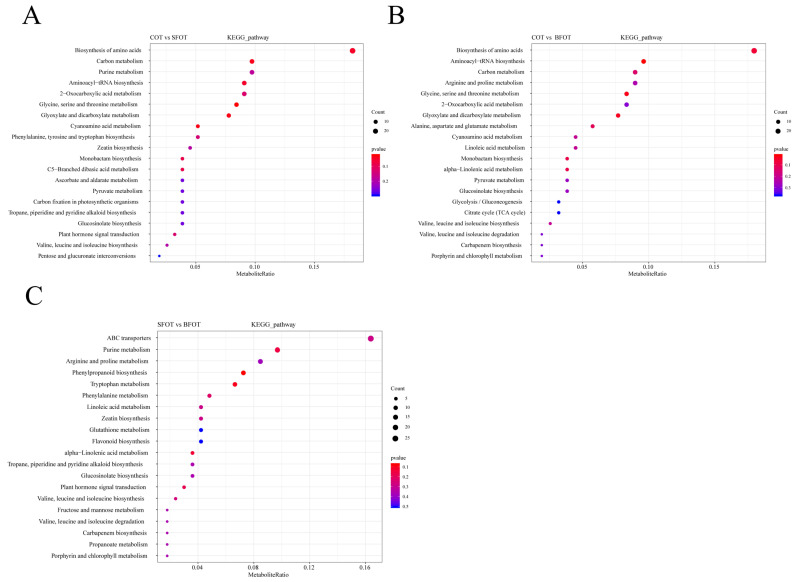
The enrichment analysis of the KEGG pathway, with the horizontal axis denoting the enrichment factor and the vertical axis indicating the pathway name. The dot color reflects the *p*-value, and the dot size corresponds to the number of differential metabolites. (**A**) KEGG pathway enrichment analysis of COT vs. SFOT; (**B**) KEGG pathway enrichment analysis of COT vs. BFOT; (**C**) KEGG pathway enrichment analysis of SFOT vs. BFOT.

In the KEGG database analysis, the majority of metabolites in the comparison between COT and SFOT were associated with key metabolic pathways, including amino acid biosynthesis, carbon metabolism, purine metabolism, aminoacyl-tRNA biosynthesis, 2-oxocarboxylic acid metabolism, glycine, serine, and threonine metabolism, and glyoxylate and dicarboxylate metabolism. These pathways are central to primary metabolism and are likely involved in fundamental biological processes such as protein synthesis, energy production, and metabolic regulation under various conditions (Figure 5A–C).

Similarly, in the comparison between COT and BFOT, metabolites were primarily linked to glyoxylate and dicarboxylate metabolism, aminoacyl-tRNA biosynthesis, carbon metabolism, arginine and proline metabolism, glycine, serine, and threonine metabolism, and 2-oxocarboxylic acid metabolism. The significant overlap of metabolic pathways observed in both comparisons suggests that these pathways play crucial roles in the metabolic adaptation and physiological responses of *C. oleifera* varieties to environmental factors. Conversely, in the comparison between SFOT and BFOT, the majority of metabolites were mapped to pathways such as the ATP-binding cassette (ABC) transporter, purine metabolism, arginine and proline metabolism, phenylpropanoid biosynthesis, and tryptophan metabolism. These pathways are notably involved in secondary metabolism, including the transport of molecules across membranes, regulation of nitrogen metabolism, and the biosynthesis of defense-related compounds. The differences in these metabolic pathways highlight potential variations in the stress tolerance and biochemical functions of the SFOT and BFOT varieties (Figure 5A–C).

## 3. Discussion

With the rapid advancement of biological sequencing technology, targeted metabolome detection methods such as UPLC-ESI-MS/MS and multiple reaction monitoring (MRM) have been established [12]. UPLC-ESI-MS/MS offers high resolution, precise characterization, short analysis time, and excellent peak separation capability, making it widely applicable for metabolomic analysis across various plant species [13,14,15]. Currently, investigations on the use of *C. oleifera* primarily focus on its seeds, which are used for edible oil production. However, limited research exists on the peel, which is restricted to the extraction of antioxidant components and determination of polyphenol content [8,9]. Therefore, this study aimed to identify metabolites in COT, BFOT, and SFOT, thereby furnishing a theoretical foundation for future production and use of *C. oleifera* pericarp.

Using FC and VIP values, 318 significantly different metabolites were identified between COT and SFOT, 449 between COT and BFOT, and 494 between SFOT and BFOT. In addition, 143 shared differential metabolites were found among the three *C. oleifera* groups. Our findings highlight flavonoids and phenolic acids as the most differentially expressed metabolites across all sample groups. These results offer valuable insights for further research on *C. oleifera* pericarp. Pericarp is recognized as having great potential as a rich source of bioactive components such as flavonoids, phenolic acids, terpenoids, and pectin, which are composed of polygalacturonic acid chains linked by α-1,4 glycosides and partially esterified with methanol. Terpenoids, which are considered modified terpenes, typically consist of isoprenoids, whereas phenolic acids have the general structure of C6–C1, and flavonoids have the C6–C3–C6 configuration; these substances exhibit the ability to scavenge free radicals [16,17,18]. Free radicals, which cause cancer and chronic diseases such as respiratory, cardiovascular, neurodegenerative, and digestive diseases, damage macromolecules such as proteins, lipids, and DNA [18]. These natural compounds can scavenge free radicals and terminate the oxidative chain reaction, thus protecting human organs [18]. Some studies have indicated that extracts of *Eriobotrya japonica* can prevent hyperlipidemia and associated toxic tissue complications [19]. Given these functionalities, the recovery of these bioactive compounds from oil tea pericarp is crucial. *C. oleifera* pericarp, which is abundant in flavonoids and polyphenolic compounds, may contribute to its hard, thick texture. Some research indicates that the polyphenolic compound gallic acid significantly influences leaf thickness and leathery texture [20,21,22].

## 4. Materials and Methods

### 4.1. Data Analysis

In this study, we analyzed the data and normalized their peak areas using R version 4.1.0 (http://www.r-project.org (accessed on 8 November 2023)) (KEGGREST and clusterProfiler for KEGG pathway enrichment analysis, ggplot2 and pheatmap for data visualization, and limma for differential metabolite expression analysis. Principal component analysis (PCA) and orthogonal partial least-squares discriminant analysis (OPLS-DA) were performed using the ropls package, while hierarchical clustering was performed using the hclust function from the stats package). The normalized data were subjected to heatmap and hierarchical analysis, PCA, and OPLS-DA (permutation tests were performed 200 times to verify the reliability of the model) analysis. Two screening criteria were employed: a fold change (FC) value of ≥2 or ≤0.5 and a VIP value of ≥1, to identify differential metabolites. The identified differentially expressed metabolites were further scrutinized using KEGG analysis [13].

### 4.2. Plant Materials

Plant materials were sourced from the *C. oleifera* nursery base of Guizhou Academy of Forestry Sciences (106°44′14.08″ E, 26°29′57.89″ N), comprising large-fruited oil tea (*Camellia semiserrata* Chi), small-fruited oil tea (*Camellia meiocarpa*), and ordinary *C. oleifera* (hereinafter referred to as BFOT, SFOT and COT, respectively) fruits collected at the mature stage (Figure 1). Three biological replicates were obtained for each *C. oleifera* variety. The selection of three trees with uniform growth was followed by the collection and mixing of four fruits from each tree to constitute one biological replicate. The chosen trees were devoid of pests and diseases. Upon fruit removal, samples were promptly placed into test tubes, flash-frozen in liquid nitrogen, and stored at −80 °C for subsequent experiments.

### 4.3. Sample Preaparation and Extraction

The samples were subjected to vacuum freeze-drying in a lyophilizer (Sci-entz-100F). This process transformed the samples into a lyophilized state. The samples were then ground using a grinder (MM 400, Retsch) at 30 Hz for 1.5 min, resulting in a fine powder. A total of 100 mg of this powder was weighed and dissolved in 1.2 mL of 70% methanol for compound extraction. The mixture was vortexed every 30 min for 30 s, with six vortex cycles in total, to ensure thorough mixing. The samples were then stored at 4 °C overnight to optimize extraction conditions. The next day, the samples were centrifuged at 12,000 rpm for 10 min, and the supernatant was separated from the precipitate. After centrifugation, the supernatant was filtered through a 0.22 μm microporous membrane to remove any particulate matter. The filtered solution was stored in an injection vial for subsequent UPLC-MS/MS analysis.

### 4.4. UPLC-MS/MS Conditions

The data acquisition instrument system primarily comprises ultra-high performance liquid chromatography (UPLC) (Agilent 1290 Infinity II UPLC, Agilent Technologies, Santa Clara, CA, USA) coupled with tandem mass spectrometry (MS/MS) (AB Sciex QTRAP 6500+ LC-MS/MS). The liquid phase conditions for UPLC are as follows [23]: the Agilent ZORBAX Eclipse Plus C18 column (2.1 mm × 100 mm, 1.8 μm particle size) was utilized. The mobile phase comprises two components: phase A, consisting of ultrapure water with 0.1% formic acid, and phase B, comprising acetonitrile with 0.1% formic acid. The elution gradient initiates at 0.00 min, with the proportion of phase B gradually increasing to 95% over 9.00 min and then maintaining a constant 95% from 10.00 to 11.10 min. Subsequently, the proportion of phase B decreased to 5% and remained stable until 14 min. The flow rate was set at 0.35 mL/min, the column temperature was set at 40 °C, and the injection volume was set at 4 μL.

### 4.5. ESI-Q TRAP-MS/MS

The mass spectrometry conditions include using an ESI Turbo V Ion Spray Interface (AB Sciex). This system is equipped with an ESI Turbo ion spray interface and can be controlled via Analyst 1.6.3 software (AB Sciex) to operate in both positive and negative ion modes. The operational parameters of the ESI source include the ion source as Turbo V spray, source temperature set at 550 °C, ion spray voltage (IS) 5500 V (positive ion mode)/−4500 V (negative ion mode), and ion source gases IGSI, GSII, and curtain gas (CUR) maintained at 50, 60, and 25.0 psi, respectively. Collision-induced ionization parameters are configured to be high. Instrument tuning and mass calibration were executed using 10 and 100 umol/L polypropylene glycol solutions in the QQQ and LIT modes, respectively. The QQQ scan operates in MRM mode with the collision gas (nitrogen) set to medium. DP (declustering potential) and CE (collision energy) optimization was performed to determine the DP and CE of each MRM ion pair. A specific set of MRM ion pairs was monitored on the basis of the eluted metabolites in each period [24].

### 4.6. Metabolite Identification and Quantification

Metabolite quantification was accomplished through multiple reaction monitoring mode (MRM) analysis employing triple quadrupole mass spectrometry [25]. In the MRM mode, the four-stage rod initially selects the precursor ions (parent ions) of the target substance to eliminate ions from other molecular weight substances, thereby reducing interference. Subsequently, the precursor ions undergo ionization in the collision chamber, yielding numerous fragment ions. These fragment ions are then filtered through the triple quadrupole to select a characteristic fragment ion, further eliminating non-target ions and improving the accuracy and reproducibility of the measurement. Following the acquisition of metabolic mass spectrum analysis data from various samples, the mass spectrometry peaks of all substances are integrated, and those of the same metabolite across different samples are also integrated and corrected. The mass spectrometry data were processed using Analyst 1.6.3 software (AB Sciex). Metabolites identification quality control is within ±5 ppm.

## 5. Conclusions

This study employed UPLC-ESI-MS/MS-based metabolic analysis to systematically compare metabolites in the pericarps of COT, BFOT, and SFOT. A total of 1117 metabolites were identified, among which 318 exhibited significant disparities between COT and SFOT, 449 between COT and BFOT, and 494 between SFOT and BFOT. In addition, 143 common differential metabolites were identified. Notably, flavonoids and phenolic acids displayed the highest abundance of differential metabolites among the three groups. Overall, this study contributes to our understanding of the metabolite composition of *C. oleifera* pericarp and offers valuable insights into its potential medical applications and future advancements.

## Figures and Tables

**Table 1:** Classification of compounds present in the fruit pericarp of the three varieties of *C. oleifera*.

Metabolite Class I	Quantity	Percentage
Flavonoids	277	24.80%
Phenolic acids	221	19.79%
Lipids	108	9.67%
Others	100	8.95%
Amino acids and their derivatives	93	8.33%
Organic acids	83	7.43%
Nucleotides and their derivatives	59	5.28%
Alkaloids	57	5.10%
Lignans and coumarins	52	4.66%
Tannins	44	3.94%
Terpenoids	23	2.06%

## Data Availability

Data is contained within the article and Supplementary Materials.

## Supplementary Materials

The following supporting information can be downloaded at: https://www.mdpi.com/article/10.3390/ijms252211973/s1.
